# In Vivo Efficacy of an Inhibitor of Complement and FcRn in Models of Glomerulonephritis and Collagen-Induced Arthritis Using Human C2 Knock-In Mice

**DOI:** 10.3390/ijms27125525

**Published:** 2026-06-18

**Authors:** Helen Cao, Amelia Nash, Yun Dai, Arthur Hsu, Amanda L. Turner, Kaushala Jayawardana, Sharon Vyas, Adele Barr, Sandra Wymann, Matthew P. Hardy

**Affiliations:** 1 CSL Ltd., Bio21 Institute, Parkville, VIC 3052, Australia; helen.cao@csl.com.au (H.C.); amelia.nash@csl.com.au (A.N.); yun.dai@csl.com.au (Y.D.); arthur.hsu@csl.com.au (A.H.); amanda.turner@csl.com.au (A.L.T.); kaushala.jayawardana@csl.com.au (K.J.); sharon.vyas@csl.com.au (S.V.); adele.barr@csl.com.au (A.B.); 2 CSL Biologics Research Centre, Swiss Institute for Translational and Entrepreneurial Medicine, 3010 Bern, Switzerland; sandra.wymann@cslbehring.com

**Keywords:** CSL305, complement, FcRn, arthritis, glomerulonephritis, autoantibody

## Abstract

A therapeutic antibody, CSL305, has been developed, which combines inhibition of the complement classical and lectin pathways via complement C2 binding with an ability to act as an antagonist of the neonatal Fc receptor (FcRn). CSL305 binds to human C2 (huC2) but shows no binding or activity against mouse C2 precluding its use in mouse models of disease to fully assess in vivo efficacy. To circumvent this, a mouse strain was developed that replaced the expression of mouse C2 with huC2 by homologous recombination. These mice (huC2 “knock-in”; KI) were shown to express huC2 protein and to have complement activity. Interestingly, male huC2-KI mice showed much stronger complement activity compared to female mice and were also sensitive to inhibition by CSL305. Two models of disease using male huC2-KI mice were then used to assess the in vivo efficacy of CSL305. The first was an attenuated passive anti-glomerular basement membrane (GBM) glomerulonephritis model involving complement activation as its primary mechanism of action. CSL305 showed dose-dependent inhibition of disease as measured by urine albumin, with reductions in kidney cellular infiltration and plasma C3 cleaved fragments C3b/C3c/iC3b also observed. The second model was a collagen autoantibody-induced arthritis (CAIA) mouse model. Here, CSL305 showed a significant and dose-dependent inhibition of clinical score in both prophylactic and therapeutic settings, mediated exclusively via its FcRn mechanism of action. Although the animal models used in this study were found to preclude the demonstration of a synergistic effect on both mechanisms, CSL305 does act in vivo as both a complement inhibitor and as a FcRn antagonist to ameliorate disease.

## 1. Introduction

The complement system plays a dual and context-dependent role in autoimmunity, functioning in immune complex clearance and host defence but, when dysregulated, acts as a powerful amplifier of inflammation and tissue injury [1,2,3]. Aberrant activation of the classical, lectin, or alternative pathways contributes to diseases such as systemic lupus erythematosus [4], anti-neutrophil cytoplasmic antibody (ANCA)-associated vasculitis [5], myasthenia gravis [6], and neuromyelitis optica spectrum disorder [7] through generation of anaphylatoxins (C3a, C5a), opsonization, membrane attack complex formation, and modulation of adaptive immunity [2,3]. This understanding has driven rapid expansion of complement-based therapeutics, extending from rare toward broader autoimmune indications [3,8,9]. Clinically validated therapeutic agents include terminal pathway inhibitors such as the anti-C5 antibodies eculizumab and ravulizumab [10,11,12], while pathway-selective upstream strategies aim to preserve host defence and reduce infectious risk. For the latter, inhibition of the classical pathway has been established with anti-C1s therapy (e.g., sutimlimab), demonstrating efficacy in antibody-mediated disease and validating proximal complement blockade as a therapeutic approach [13,14]. More recently, Argenx developed an anti-C2 mAb, Empasiprubart/ARGX-117 as a means of simultaneously suppressing classical and lectin pathway activation, with preclinical and early translational studies highlighting its potential to limit immune-complex-driven inflammation while sparing the alternative pathway [15,16,17]. In parallel, small-molecule strategies such as the oral C5a receptor antagonist avacopan have validated selective effector blockade in ANCA-associated vasculitis [18,19]. Collectively, these advances underscore a shift toward precision complement modulation—targeting defined nodes including C1s, C2, C3, C5, and C5aR1—positioning complement therapeutics as a rapidly maturing and increasingly nuanced platform for the treatment of autoimmune diseases.

FcRn plays a central role in autoimmunity by regulating the homeostasis, recycling, and transcytosis of immunoglobulin (Ig) G, thereby prolonging the half-life of both protective and pathogenic antibodies [20,21]. In IgG-mediated autoimmune diseases, FcRn-dependent rescue of autoantibodies sustains immune complex formation, Fc gamma receptor engagement, and downstream inflammatory cascades, providing a strong mechanistic rationale for therapeutic FcRn blockade [20,21,22]. Pharmacologic inhibition of FcRn accelerates lysosomal degradation of circulating IgG without broadly suppressing immune cell function, offering a targeted means of reducing pathogenic autoantibody burden [20]. This strategy has been clinically validated with several FcRn antagonists, most notably efgartigimod, rozanolixizumab, and nipocalimab, which have demonstrated rapid, dose-dependent reductions in total IgG and clinically meaningful efficacy in antibody-mediated diseases [23,24,25]. Efgartigimod and rozanolixizumab are now approved for generalised myasthenia gravis, while additional agents such as nipocalimab and batoclimab are advancing across indications including immune thrombocytopenia, warm autoimmune haemolytic anaemia, and systemic rheumatic diseases [23,24,25,26]. Collectively, these data establish FcRn antagonism as a clinically validated, mechanism-based approach for selectively modulating humoral autoimmunity, complementary to B-cell-directed or complement-targeted therapies.

CSL305 is a human monoclonal antibody (mAb) engineered so that its fragment antigen binding (Fab) region binds C2 zymogen and active C2b at its catalytic cleft and thereby inhibits complement classical and lectin pathway activity whilst leaving the alternative pathway intact [27]. The addition to its fragment crystallizable (Fc) region of a triple mutation that increases affinity to FcRn at both acidic and neutral pH allows CSL305 to act as a dual antagonist of both complement and FcRn and therefore is of potential clinical value. Data from cynomolgus monkeys showed a significant (>50%) and prolonged duration of ex vivo classical pathway inhibition following CSL305 administration and a reduction in endogenous IgG levels, consistent with the mAb acting on both complement and IgG recycling [27]. Pharmacokinetic data also showed a rapid clearance of CSL305 in plasma, indicating the retention of CSL305 in a cell-associated context, which allowed it to retain potency for an extended duration. The use of mice in these studies allowed interrogation of changes in endogenous IgG levels by CSL305 but precluded an analysis of complement inhibition due to the lack of cross-reactive binding to mouse C2, a significant drawback for further analysis in animal models of disease where both mechanisms of action would need to be investigated to demonstrate its dual functionality.

In this study, we overcome the lack of animal cross-reactivity of CSL305 by generating a huC2-KI mouse strain so that both complement inhibition and IgG recycling (via FcRn antagonism) can be explored in the context of autoimmune models of disease. We show the dose-dependent efficacy of CSL305 in both an anti-GBM glomerulonephritis and a CAIA mouse model using huC2-KI mice to demonstrate that this mAb can inhibit each of its mechanisms of action in vivo.

## 2. Results

### 2.1. Generation and Characterisation of huC2-KI Mice

**Figure 1:**
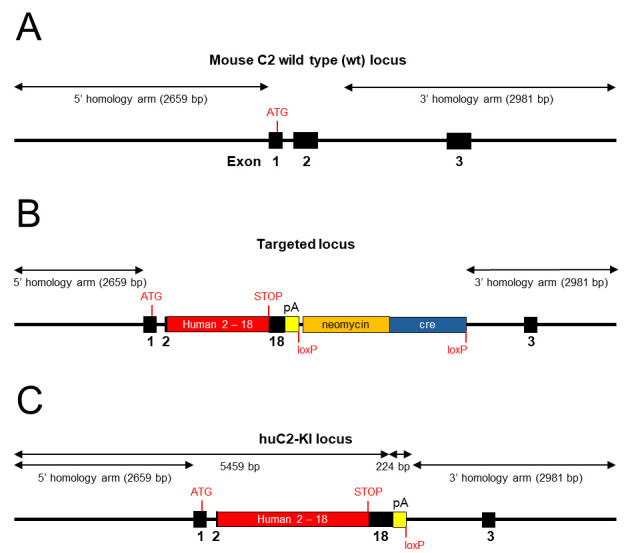
**Targeting strategy to generate human C2 knock-in (huC2-KI) mice.** (**A**) The mouse C2 gene locus is shown as a horizontal black line with the positions of mouse exons denoted as black boxes. The position of the initiating methionine (ATG) is indicated on exon 1, and the 5′ and 3′ homology arms with sizes (base pairs; bp) used as part of the targeting strategy are shown as double-ended arrows. (**B**) Mouse C2 locus with the following insertions: exons 2–18 of the human C2 gene as a single cDNA (red box) encoding A21-L752 of huC2, fused to mouse exon 2. This is followed by a STOP codon, then exon 18 of the mouse C2 gene containing the mouse C2 3′ untranslated region, and a polyadenylation sequence (pA; yellow box). Also inserted is a neomycin cassette (orange box) for selection of embryonic stem (ES) cells and loxP recognition sequence for cre recombinase (blue box) mediated neomycin removal. (**C**) Gene-targeted huC2-KI locus after cre-mediated neomycin removal. Expression of mouse C2 is lost due to disruption of the mouse C2 gene and is replaced by the expression of the mature huC2 sequence encoded by exons 2–18 of the human C2 gene. Nucleotide fragment sizes across the huC2-KI locus are denoted in base pairs (bp).

**Figure 2:**
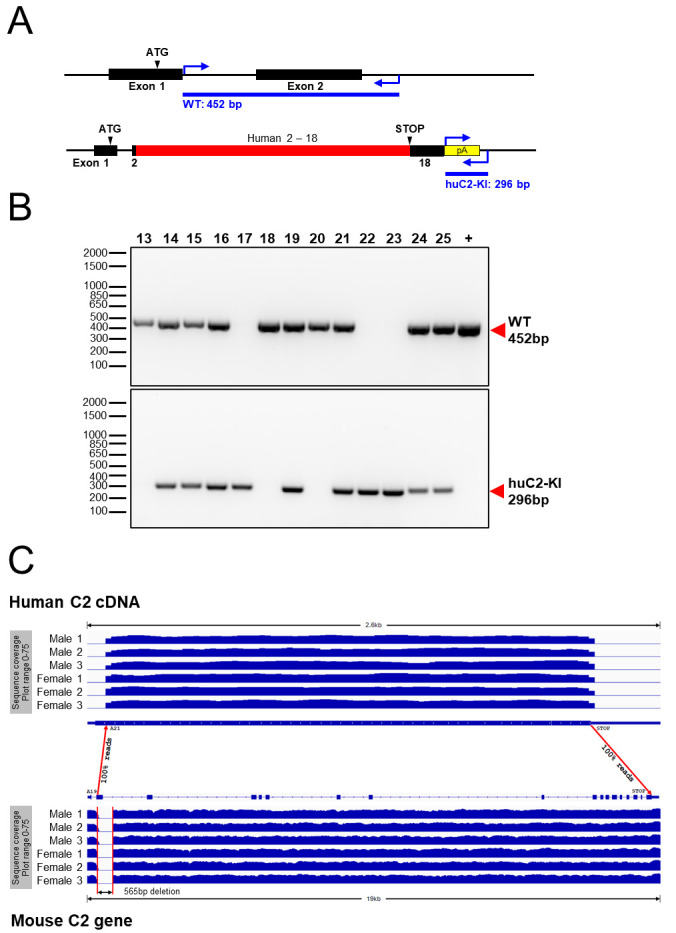
**Screening of huC2-KI mice.** (**A**) Screening strategy for identification of wild-type (top) or huC2-KI (bottom) mice, using the schema of the wild-type and targeted loci, respectively (not to scale). Location and direction of primers used for detection of relevant PCR fragments (blue bar) are denoted by arrows. (**B**) Animals were screened for wild-type (WT) and hC2-KI alleles through PCR and the products detected through agarose gel electrophoresis, with 452 bp or 296 bp products denoting the presence of the WT or huC2-KI alleles, respectively. A control C57BL/6J mouse sample (+) and molecular weight markers, indicated in base pairs (bp), were included. (**C**) Whole-genome sequencing data from three male and three female mice showing the successful knock-in (see red arrows) of the human C2 cDNA sequence (top; refer to Ensembl transcript ID ENST00000299367.10) into the murine C2 locus (bottom; refer to Ensembl transcript ID ENSMUST00000025230.15) with 100% of the reads supporting the integration. The panel depicts a C2 DNA sequence that starts with the murine C2 5′ untranslated region until the codon encoding amino acid 19 (A19), then switching to the human C2 coding sequence from amino acid 21 (A21) until the STOP codon (STOP), followed by a switchback to the murine C2 3′ untranslated region. The 565 bp homozygous deletion of the endogenous murine C2 exon 2 is also shown, ensuring the disruption of mouse C2 expression. Each blue row shows sequence coverage for each mouse sequenced. The human cDNA schema is shown below the top panel and the mouse gene with introns and exons depicted is shown above the bottom panel. The relative sizes, in kilobase pairs (kb), are shown for each panel as a black horizontal line.

To enable the in vivo assessment of CSL305 in mouse models of disease, we sought to replace mouse C2 with huC2 using a knock-in (KI) targeting strategy. To this end, the wild-type (WT) mouse C2 locus (Figure 1A) was targeted for insertion of exons 2–18 of huC2 as a single complementary DNA (cDNA) into exon 2 of the mouse C2 gene. At the 3′ end a STOP codon was added, followed by exon 18 of the mouse C2 gene encoding the 3′ untranslated region, a polyadenylation sequence, a neomycin cassette, and loxP recognition sequences for cre recombinase-mediated neomycin removal (Figure 1B). The huC2-KI locus is shown in Figure 1C. Screening for WT mouse or huC2-KI alleles was then undertaken by polymerase chain reaction (PCR) (Figure 2A), with several huC2-KI mouse lines on a C57BL/6J background (numbers 17, 22, 23) identified (Figure 2B). As no reliable mouse-specific C2 assay was available to confirm the deletion of mouse C2 at the protein level, we turned to a genomic sequencing approach to demonstrate the disruption of the mouse C2 gene and to confirm the correct insertion of the huC2 cDNA in the huC2-KI mice. The results are shown in Figure 2C where the correct insertion of the cDNA encoding huC2 into the mouse C2 locus is indicated.

**Figure 3:**
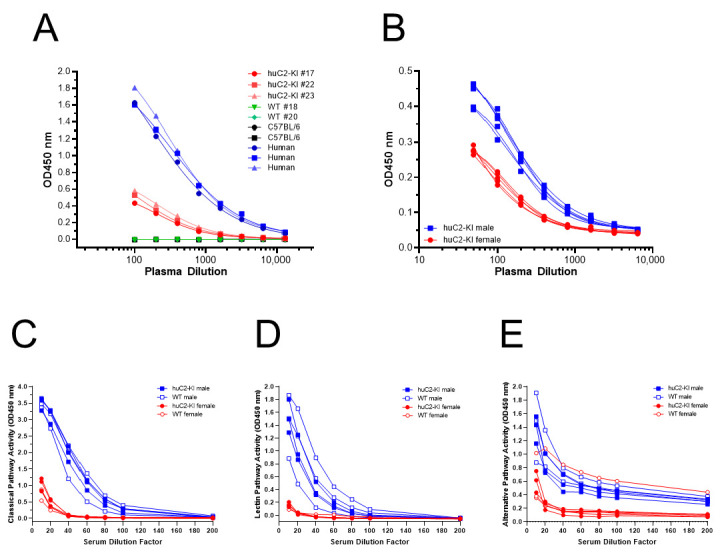
**Human C2 expression and complement activity in huC2-KI mice.** (**A**) Human C2 detected in serially diluted plasma samples taken from individual animals from selected WT or huC2-KI mouse lines (see Figure 2B) by ELISA. Three separate representative human and two separate C57BL/6J mouse plasma samples were used as positive and negative controls, respectively. (**B**) Plasma samples from individual WT and huC2-KI male and female mice were then comparatively assessed for human C2 expression at a range of plasma dilutions (*N* = 5 mice each); serum samples were analysed for (**C**) complement classical pathway, (**D**) lectin pathway, and (**E**) alternative pathway activity using mouse Hycult kits specific for each pathway at a range of serum dilutions (1/10 to 1/200; *N* = 3 each). Activity is expressed graphically for individual animals as optical density (OD) at 450 nm for each serum dilution factor employed.

The homozygous huC2-KI mice thus generated were then assessed for expression of huC2 protein through enzyme-linked immunosorbent assay (ELISA). The samples from naive C57BL/6J and from WT mice identified from the PCR screening protocol were included as negative controls, whereas separate human plasma samples served as positive controls. The results are shown in Figure 3A. No huC2 expression was measured in WT or naive C57BL/6J mice. In contrast all human plasma and huC2-KI mouse samples showed huC2 expression, although expression in huC2-KI mouse samples was lower in all plasma dilutions tested. Given previously noted sex differences in the level of complement proteins and complement activity in mice [28], an assessment of sex-based differences in huC2 expression was then undertaken in huC2-KI mice. The huC2 ELISA results showed lower huC2 expression levels in female huC2-KI mice compared to male huC2-KI mice in all plasma dilutions tested (Figure 3B). This was followed by a comparative assessment of complement pathway activity in WT and huC2-KI mice. As shown in Figure 3C, serum samples from female WT and huC2-KI mice both showed low classical pathway activity compared to samples from male mice, with no difference in activity observed between WT and huC2-KI mice of each sex. A similar result was obtained for the complement lectin (Figure 3D) and alternative pathway (Figure 3E). This data confirms that sex-based differences in complement activity are also present in huC2-KI mice, regardless of the presence of huC2.

**Figure 4:**
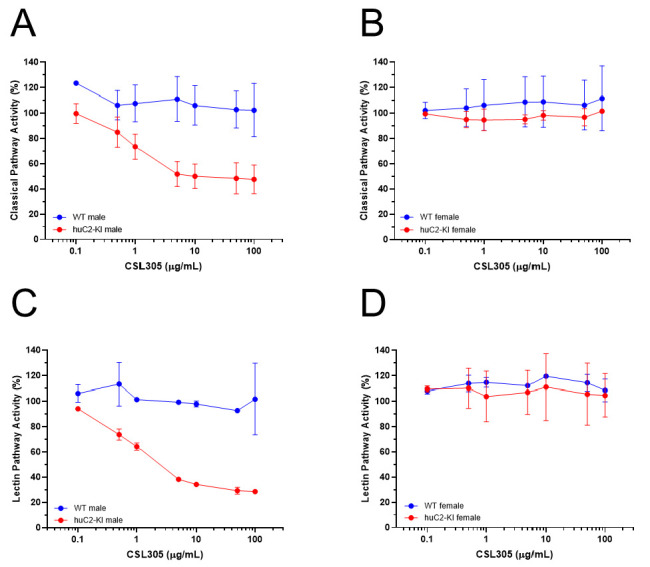
**Dose-dependent complement inhibitory activity of CSL305 in vitro.** CSL305 was tested in classical pathway (**A**,**B**) and lectin pathway (**C**,**D**) assays, using serum from WT or huC2-KI male (**A**,**C**) or female (**B**,**D**) mice. Data points show the mean ± SD pathway activity (%, *N* = 3) for each CSL305 concentration (μg/mL) tested.

To further demonstrate the functionality of huC2 expression in huC2-KI mice and to address their suitability for in vivo efficacy studies, CSL305 was assessed for its ability to inhibit serum complement pathway in vitro. As shown in Figure 4A, CSL305 was able to dose-dependently inhibit classical pathway activity in samples taken from male huC2-KI, but not male WT mice, demonstrating the specificity of CSL305 for human, but not mouse C2. Interestingly, no inhibition of classical pathway activity was observed in female huC2-KI mice (Figure 4B), suggesting that the low pathway activity shown above may be compromising their suitability for inhibition studies. Likewise, CSL305 was able to dose-dependently inhibit lectin pathway activity in male huC2-KI but not WT mice (Figure 4C) or in female mice (Figure 4D). Taken together, this data indicated that male, but not female huC2-KI mice, were suitable for further assessment in in vivo models of the disease.

### 2.2. CSL305 Reduces Injury in an Anti-GBM Glomerulonephritis Model

**Figure 5:**
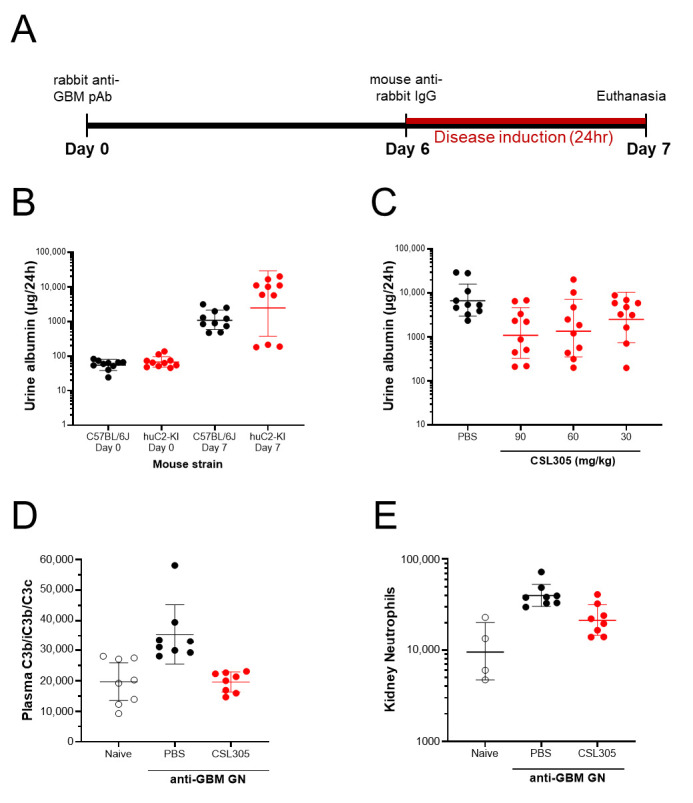
**Establishment of the anti-GBM glomerulonephritis model in huC2-KI mice and efficacy assessment of CSL305.** (**A**) Model set up: glomerulonephritis was induced in male C57BL/6J or huC2-KI mice through IV injection of rabbit anti-GBM polyclonal antibody, which was followed 6 days later by IP administration of mouse anti-rabbit antibody. Urine is collected using metabolic cages for 24 h until sacrifice at day 7. Additional samples are taken during this period for biomarker assessment. (**B**) Urine albumin concentration in C57BL/6J and huC2-KI male mice at day 0 (non-disease controls) and day 7, 24 h after mouse anti-rabbit antibody administration. *N* = 10 mice per group. (**C**) Urine albumin concentrations taken at day 7 from huC2-KI male mice treated with PBS or CSL305 at 30, 60, or 90 mg/kg (administered IP 1 h prior to the anti-rabbit antibody). *N* = 10 mice per group. (**D**) Effect of CSL305 (90 mg/kg) treatment on the plasma levels of complement activation fragments C3b/iC3b/C3c after 1 h induction of glomerulonephritis in a separate study. Levels in naïve huC2-KI male mice (non-disease control) were used as a negative control. *N* = 8 mice per group. (**E**) Infiltrating neutrophils in kidney quantified through flow cytometry using Ly6G and CD11b markers, from *N* = 4 naïve, *N* = 8 PBS-treated and *N* = 8 CSL305-treated (90 mg/kg) huC2-KI mice after 3 h induction of glomerulonephritis. Data are expressed as geometric mean ± geometric SD (**B**,**C**,**E**) or mean ± SD (**D**). Group differences were evaluated using a non-parametric Kruskal–Wallis test (panel **B**,**D**) or one-way ANOVA (panel **C**,**E**), with post hoc comparisons performed using Dunn’s test (panel **B**–**D**) or Tukey’s test (panel **E**).

The attenuated passive anti-GBM glomerulonephritis mouse model was selected to assess the complement inhibitory potential of CSL305 in vivo. The model (Figure 5A), which uses immune complexes directed against the GBM to induce a mild but acute kidney disease over a 24 h period, has a significant complement component to it and no FcRn involvement [29,30]. Comparative assessment of disease induction, as measured by urine albumin content, was first performed using WT C57BL/6J and huC2-KI male mice. As shown in Figure 5B, a significant increase (*p* < 0.005) in urine albumin was measured at the end of the study period on day 7 compared to non-diseased animals from the same strain at day 0, with no significant differences observed between WT and huC2-KI mice. A dose response experiment was then conducted with CSL305 administered intraperitoneally to huC2-KI male mice at 30, 60 or 90 mg/kg and urine albumin measured against a phosphate-buffered saline (PBS)-treated control group. Significant reductions in urine albumin were observed in the 90 mg/kg (*p* < 0.01) and 60 mg/kg (*p* < 0.05) groups compared to control animals (Figure 5C). No significant difference was measured for the 30 mg/kg CSL305 group. The effect of CSL305 on systemic complement activation was assessed using C3b/C3c/iC3b ELISA at the highest dose of 90 mg/kg. It was determined that C3b/C3c/iC3b levels were significantly increased (*p* < 0.005) 1 h following from the start of disease induction compared to non-diseased naïve animals (Figure 5D); at this timepoint CSL305 was able to significantly reduce (*p* < 0.005) this back to the levels found in naive huC2-KI mice. Renal neutrophil infiltration was also assessed at its peak at 3 h following disease induction [30], where a significant increase (*p* < 0.0005) in kidney neutrophils was measured between the naive and PBS control groups. CSL305 administration was able to significantly reduce these neutrophil numbers (*p* < 0.05) compared to the PBS control group (Figure 5E). Taken together, this data shows that CSL305 is an effective inhibitor of complement-mediated anti-GBM glomerulonephritis in vivo.

### 2.3. CSL305 Ameliorates Disease in the Collagen Autoantibody-Induced Arthritis (CAIA) Model

**Figure 6:**
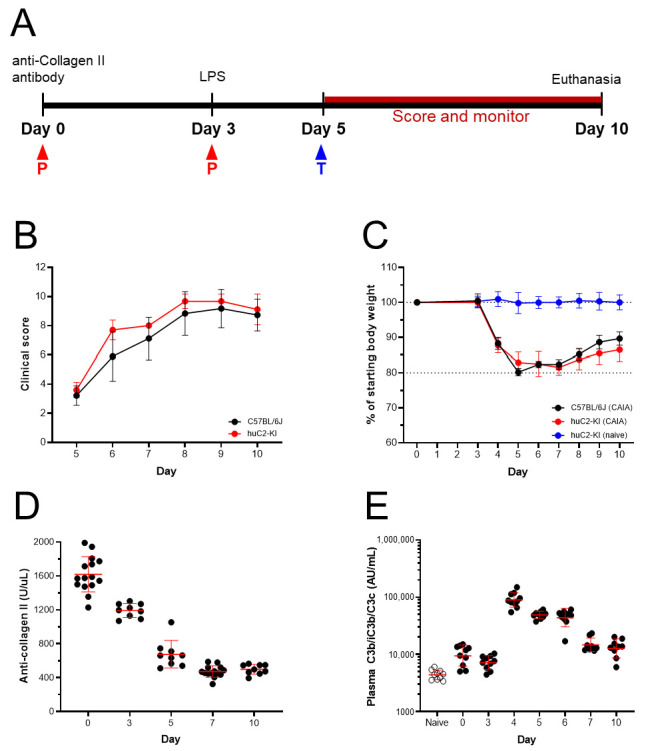
**Establishment of the CAIA model in huC2-KI mice.** (**A**) Model set up. Arthritis is induced in male C57BL/6J or huC2-KI mice through IP injection of 2 mg anti-collagen II antibody cocktail followed at day 3 through IP administration of 50 µg LPS. Disease scoring was between days 5 and 10. IP administration of CSL305 prophylactically (P) on days 0 and 3 or therapeutically (T) on day 5. (**B**) Clinical scoring of arthritis progression in the CAIA model in either male C57BL/6J (*N* = 9) or huC2-KI (*N* = 10) mice between days 5–10. Scoring was conducted in a blinded manner on all 4 paws. (**C**) Percent of starting body weight over time in C57BL/6J (*N* = 9) and huC2-KI (*N* = 10) mice over the course of the CAIA model. Naïve huC2-KI mice (*N* = 10) were used as a negative control (no disease induction). Dotted lines are set at 80% and 100% starting body weight. (**D**) Anti-collagen II levels (U/µL) measured from samples taken from huC2-KI mice at day 0, 3, 5, 7, 10 (*N* = 15, 9, 9, 14, 9, respectively). (**E**) Plasma C3b/iC3b/C3c levels (AU/mL) measured from samples taken from mice at day 0, 3, 4, 5, 6, 7, 10 (*N* = 10, 10, 10, 9, 10, 8, 9, respectively) plus naïve control mice (*N* = 10). Data are expressed as mean ± SD. Statistical analyses were performed using: a linear mixed effects model with Šídák’s multiple comparison test (panel **B**,**C**), one-way ANOVA with Dunnett’s multiple comparisons test (panel **D**), or a non-parametric Kruskal–Wallis test followed by Dunn’s multiple comparisons test (panel **E**).

We next turned to the CAIA mouse model, a second antibody-driven, immune-complex-mediated inflammatory disease model previously shown to have both complement and FcRn involvement [31,32], to determine whether CSL305 could inhibit both complement- and FcRn-mediated injury. Induction of arthritis in this model relies on the administration of an anti-collagen II mAb (anti-CII) cocktail to mice at day 0, followed by lipopolysaccharide (LPS) administration at day 3 to boost disease severity, with daily disease scoring from day 5 to peak disease at day 10 (Figure 6A). Disease progression, as measured by clinical scoring of arthritis in the limbs, was first compared between male WT C57BL/6J mice and huC2-KI mice to determine the suitability of the latter strain for assessment of CSL305. Figure 6B shows no significant difference in the progression of disease between these groups. During the study period, there was also a drop in body weight in diseased mice compared to naive mice, peaking at day 5 before recovering; no differences were observed between WT and huC2-KI mouse groups (Figure 6C). An analysis of the plasma anti-CII antibody concentrations in huC2-KI mice following disease induction revealed a steady and significant decrease (*p* < 0.0001) after anti-CII mAb administration on day 0 to days 7 and 10 where the measured concentrations stabilised (Figure 6D). As there was no available information pertaining to the kinetics of complement activation throughout the disease course of the CAIA model, the activity of the complement activation marker plasma C3b/iC3b/C3c was measured. As shown in Figure 6E, levels were slightly elevated on day 0 (2 h following anti-CII administration; *p* < 0.0001) and day 3 (2 h following LPS administration; *p* < 0.005). Complement activation peaked at day 4 where significant increases were observed, which slowly decreased toward the end of the study period (days 4–6: *p* < 0.0001; days 7, 10: *p* < 0.05 for huC2-KI mice with CAIA compared to naive controls).

**Figure 7:**
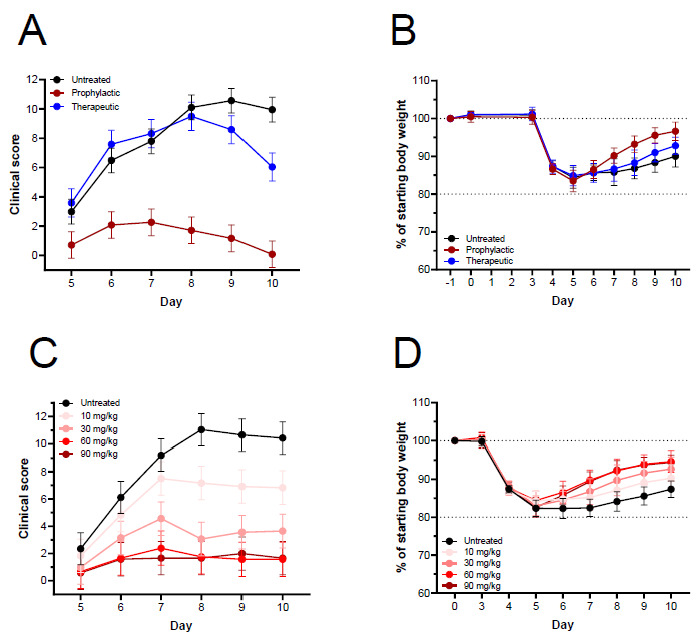
**Effect of CSL305 in the CAIA model.** (**A**) Comparative analysis of CSL305 administered IP to male huC2-KI mice in a therapeutic setting at 90 mg/kg on day 5 (*N* = 11) or prophylactically at days 0 and 3 (90 mg/kg; *N* = 11), with untreated mice used as controls (*N* = 13). Clinical score (conducted blinded) from days 5 to 10, inclusive, is shown. (**B**) Body weight assessment of the same animals used in panel A, measured as the percentage of starting body weight on indicated days. Dotted lines are set at 80% and 100% starting body weight. (**C**) Dose response study of CSL305 in the prophylactic setting, with doses of 10, 30, 60 and 90 mg/kg (*N* = 12 mice per group) administered IP and a control, untreated group also used (*N* = 13). (**D**) Body weight assessment of the same animals used in panel C. Data are expressed as estimated marginal means with 95% Confidence Intervals (**A**,**C**) or mean ± SD (**B**,**D**). Statistical analyses were performed using a linear mixed effects model with Dunnett’s multiple comparison test against untreated controls (**A**–**D**).

CSL305 was then tested in the CAIA model in male huC2-KI mice in both prophylactic and therapeutic settings. In one group, 90 mg/kg CSL305 was administered prophylactically at day 0 and again at day 3; a second therapeutic treatment group had the same dose administered at day 5 only (Figure 7A). The results show that compared to an untreated group of mice, prophylactic administration of CSL305 caused a significant reduction in clinical score (*p* < 0.01 at day 5; *p* < 0.0001 for days 6–10) across the entire assessment period with almost complete amelioration of disease by day 10 (Figure 7A). Therapeutic administration of CSL305 at day 5 also showed some improvement in clinical score, but only at day 9 (*p* < 0.01) and day 10 (*p* < 0.0001; Figure 7A). There was a concomitant improvement in mouse body weight observed with CSL305 treatment compared with untreated huC2-KI mice with CAIA, with a significant benefit measured following prophylactic treatment (day 7: *p* < 0.005; days 8–10: *p* < 0.0001; Figure 7B). A dose response study of CSL305 using the same prophylactic treatment protocol as above was then conducted. The data showed a benefit of CSL305 treatment compared to untreated disease controls at doses as low as 10 mg/kg at days 8–10 (*p* < 0.001), with significant improvements in clinical score observed at 30, 60 and 90 mg/kg (*p* < 0.0001 for all three doses at days 6–10 except *p* < 0.01 for 30 mg/kg at day 6; Figure 7C). A dose-dependent and significant improvement in body weight over the course of the study period for treated compared to untreated animals was also observed (day 6: *p* < 0.01 for 60, 90 mg/kg; day 7–8: *p* < 0.01 for 30 mg/kg and *p* < 0.0001 for 60, 90 mg/kg; day 9: *p* < 0.05 for 10 mg/kg and *p* < 0.0001 for 30, 60, 90 mg/kg; day 10: *p* < 0.0001 for 30, 60, 90 mg/kg; Figure 7D).

**Figure 8:**
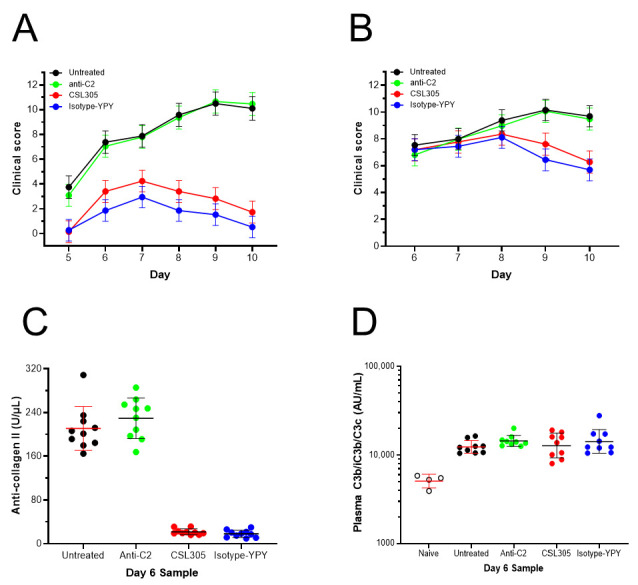
**Mechanistic assessment of CSL305 in the CAIA model.** Comparative analysis of CSL305 administered to male huC2-KI mice at in (**A**) a prophylactic setting on day 0 and day 3 at 30 mg/kg, and (**B**) therapeutic setting on day 5 at 90 mg/kg, with the following control groups at the same doses: untreated; anti-C2 mAb; and isotype mAb with an Fc YPY mutation. *N* = 12 mice/group, except untreated for (**B**), which was *N* = 13. The clinical score between days 5 (**A**) or 6 (**B**) and 10, inclusive, is shown graphically. Day 6 samples from the prophylactic study were also assessed for (**C**) anti-collagen II concentrations (U/µL; *N* = 10 randomly selected) and (**D**) plasma C3b/iC3b/C3c concentrations (AU/mL; *N* = 9 randomly selected with an additional *N* = 4 samples from naïve animals as a further control). Data are expressed as estimated marginal means with 95% Confidence Intervals (**A**,**B**) or mean ± SD (**C**,**D**). Statistical analyses were performed for panels **A** and **B** using a linear mixed effects model with Dunnett’s multiple comparison test against untreated controls. For panels **C** and **D**, a one-way ANOVA with Dunnett’s multiple comparison test was used.

In order to understand the contribution of the complement and/or FcRn mechanisms of CSL305 on the CAIA disease amelioration observed above, a subsequent study compared additional single mechanism control group arms with CSL305. A mid-efficacious dose of 30 mg/kg CSL305 was selected using the prophylactic treatment protocol to highlight efficacy differences against equimolar doses of an anti-C2 mAb (the Fab region identical to CSL305) and an isotype mAb FcRn antagonist control (containing the same FcRn-modifying mutations—the “YPY” motif-on the Fc region as CSL305 [27]). As shown in Figure 8A, the isotype control FcRn antagonist showed similar efficacy in the CAIA model as CSL305 compared to untreated controls (*p* < 0.0001 for days 5–10). However, no significant difference in disease score was observed between the anti-C2 mAb control-treated and untreated control animals, suggesting that the efficacy of CSL305 was due purely to its FcRn antagonist mechanism of action with no contribution of its complement inhibitory arm. A similar experiment was performed using the therapeutic protocol with all mAbs administered at 90 mg/kg. The results (Figure 8B) confirmed that the efficacy of CSL305 was solely due to FcRn antagonism, with significant reduction in disease scores from CSL305- and YPY-contained isotype control-treated animals compared to anti-C2 and untreated animals (*p* < 0.0001 at days 9–10; all other time points not significant). To provide a mechanistic explanation for the clinical scores observed, day 6 samples from the CSL305 prophylactic study shown in Figure 8A were used to assess the effect of CSL305 on anti-collagen type II antibody levels and plasma C3b/iC3b/C3c concentrations. Figure 8C shows that, in day 6 samples from both CSL305- and isotype control FcRn antagonist-treated huC2-KI mice, and collagen type II antibody levels were significantly lower compared to untreated and anti-C2 mAb treated animals (*p* < 0.0001), which were not significantly different from each other. In contrast, Figure 8D shows no significant reduction in plasma C3b/iC3b/C3c concentrations in any of the day 6 samples taken from all treatment groups. These results further suggest that the FcRn antagonistic effects but not C2 inhibition of CSL305 are responsible for the amelioration of the clinical scores observed.

## 3. Discussion

Finding a suitable animal model to demonstrate the in vivo efficacy of a given therapeutic can often be challenging, the more so for CSL305 since its complement inhibitory and FcRn antagonistic functions are contained within the one molecule. An additional complication observed with CSL305 was the lack of cross-reactive binding and activity towards C2 from species other than humans and non-human primates [27]. This made any assessment of the in vivo efficacy of CSL305 problematic, since many autoimmune-related animal models of disease that have both a significant complement and a pathogenic autoantibody component to them are confined to rodent species.

To overcome the problem of appropriate cross-reactivity for CSL305, we generated a huC2-KI mouse line where mouse C2 was replaced with huC2. Expression of huC2 in KI mice was observed to be lower than endogenous huC2 measured from human serum samples, which can be explained by the retention of the mouse C2 promoter within the targeting construct (Figure 1) and reports of significantly lower complement activity in mice compared to humans [33]. Reference ranges for mouse C2 found in commercially available kits are between 50 and 300 ng/mL, in contrast to huC2 levels measured from clinical samples at the higher range of 4–40 µg/mL [34]. It was much more difficult to demonstrate the absence of mouse C2 expression in KI animals brought about by the insertion of the huC2 transgene, as no reliable mouse C2-specific assays were available. However, the use of whole-genome sequencing convincingly demonstrated the disruption of the mouse C2 gene by the appropriate targeting of the huC2 expression cassette in exon 2 of mouse C2. A decrease in huC2 levels was also observed in female KI mice compared to male mice, which was mirrored by low complement activity for all pathways (Figure 3). The much more robust complement activity in male compared to female KI is consistent with previously reported sex-based differences in complement component protein levels and activity, where female mice showed significantly lower activity for all three complement pathways as well as C2 and the terminal complement components C6 and C9. A convincing explanation for this phenomenon in mice has been suggested to be due to the effects of sex hormones on levels of terminal complement components with early studies showing significant effects of castration and exogenous administration of testosterone and oestrogen on terminal complement activity in male and female mice [28,35,36,37]. These differences are not found anywhere near to the same extent in the human population, where, for example no differences in huC2 levels and for classical complement pathway in general have been observed between men and women [38]. Lower complement alternative pathway activity and levels of terminal complement components C5 and C8 were measured in healthy females compared to males, however, highlighting species-specific differences in complement activity. It is interesting that a clinical study involving the complement inhibitor TP10 did show sex-specific differences with only males showing a significant clinical response [39].

Due to low complement pathway activity in female huC2-KI mice, CSL305 was only able to inhibit complement classical and lectin pathway activity in male huC2-KI mice. Because of these findings, we used male huC2-KI mice exclusively for all subsequent in vivo experiments. No inhibition of complement classical or lectin pathway was measured for CSL305 in WT mice, demonstrating the specificity of its activity to huC2 in huC2-KI mice; CSL305 has previously been shown to have no effect on alternative pathway activity [27]. Although a dose-dependent inhibition of complement classical and lectin pathway activity in huC2-KI male mice in vitro by CSL305 was observed, complete inhibition could not be achieved. The high serum dilutions required for pathway specificity in these assays are a possible explanation for the incomplete inhibition observed given the phenomenon is well described and similar findings have also been noted with other complement inhibitors using this type of assay [40,41]. Incomplete target engagement or a ceiling effect of the drug’s efficacy also cannot be entirely ruled out.

Although not strictly speaking an autoimmune disease model, the attenuated passive anti-GBM glomerulonephritis mouse model mechanistically remains both antibody-dependent and Fc gamma receptor- and complement-driven [29], and thus, as an immune-complex-mediated model, was deemed suitable to begin exploring the in vivo efficacy of CSL305. A caveat was that the model is acute with peak kidney neutrophil infiltration present at 3 h after disease induction and urine albumin measured at 24 h, which made it unsuitable to assess FcRn antagonism. Therefore, only the complement mechanism of action of CSL305 was assessed. There was some uncertainty about the level of complement alternative pathway involvement in this disease model, but the dose-dependent amelioration of disease with CSL305 (Figure 5) clearly suggest a significant contribution of the classical and/or lectin pathway. Not only was there an attenuation of urine albumin, but plasma C3b/iC3b/C3c fragment levels and the number of kidney neutrophils were also reduced.

The CAIA mouse model that was also used in this study is likewise an antibody-transfer, immune-complex-mediated inflammatory model that reproduces the effector phase of autoimmune arthritis but not the adaptive immune initiation phase [42]. The strength of evidence for complement involvement in this model is strong [31,42,43], while disease magnitude and persistence are critically regulated by FcRn-dependent IgG recycling [32,44]. This evidence suggested that the CAIA model was suitable to explore both mechanisms of action in the context of CSL305. As part of our model establishment, we showed that the huC2-KI mice developed arthritis to the same degree as wild-type control mice, with a gradual decrease in anti-CII mAb levels following administration and an increase in plasma C3b/iC3b/C3c levels following anti-CII mAb and LPS boost, which tapered towards the end of study at day 10 when the arthritis clinical score had peaked (Figure 6). CSL305 proved to be an effective and dose-dependent inhibitor of disease in the CAIA model as measured by clinical scores, particularly when administered prophylactically (Figure 7), although significant efficacy in a therapeutic setting was also observed. However, when further experiments were conducted, including single arm mechanistic controls (anti-C2 only; anti-FcRn only) to tease out the contribution of complement and/or FcRn antagonism to the amelioration of disease (Figure 8), it became clear that the entire disease inhibitory response by CSL305 was mediated via FcRn antagonism and that inhibition of the complement classical and/or lectin pathway did not significantly alter disease progression. It is suggested, therefore, that, despite the initiation of the complement classical pathway by IgG-collagen II immune complexes in CAIA models incorporating LPS as a secondary stimulus as previously described [31,45,46], disease induction is critically dependent on alternative pathway amplification [45] and inhibition of the classical pathway does not significantly affect disease progression. A previous study in the same model also showed that selective inhibition of the complement alternative pathway using a CR2-factor H fusion protein attenuated disease progression [47]. Despite demonstrating that the FcRn antagonistic capability of CSL305 can ameliorate disease, the CAIA model was found to be not suitable for assessing both mechanistic arms simultaneously, and further models will need to be explored to address this question.

## 4. Materials and Methods

### 4.1. Generation of C2 Non-Conditional Knock-In Humanised Mice

The generation of these mice was conducted at Ozgene (Perth, Australia). A non-conditional knock-in humanised mouse model of the C2 gene was generated by knocking human cDNA of the 752-residue human C2 isoform 1 (UniProt: P06681-1) into exon 2 of transcript ENSMUST00000025230.15 of the murine C2 gene. This was achieved by fusing mouse exon 2 at A19 in mouse with human cDNA exons 2–18 (Ala21 to Leu752) till the STOP codon, inserting the murine 3′ untranslated region (UTR) straight after the STOP codon, and including an exogenous BGH polyadenylation (pA) and a loxP flanked neomycin cassette downstream of the 3′ UTR. The genetic modification was introduced into Bruce4 C57BL/6 [48]. Correctly targeted embryonic stem cell clones were identified and then injected into goGermline [49]. Male goGermline^TM^ mice were bred to C57BL/6J females to establish heterozygous germline offspring on a C57BL/6J background. Homozygous mice were generated from additional breeding and used in all experiments.

### 4.2. Genotyping

Mouse ear clip samples were digested in DirectPCR solution (Viagen Biotech, Los Angeles, CA, USA), with 200 μg/mL Proteinase K (Promega, Sydney, Australia) added, at 55 °C overnight. Proteinase K was then inactivated at 85 °C for 45 min. For PCR genotyping, a WT and a KI reaction was run with DNA template (2 μL) amplified in a 25 μL reaction with the primers: WT forward 5′-GAG GCT GGG AAA GGG GTT ATT G-3′; WT reverse 5′-CCT GGA GAA CCC ACT GCT AAT AAG-3′; huC2-KI forward 5′-TAG TTG CCA GCC ATC TGT TGT TTG-3′; and huC2-KI reverse 5′-GAT CTG GAC GAA GAG CAT CAG G-3′. The following PCR conditions were used—35 cycles of 95 °C, 30 s; 59.5 °C, 30 s; and 72 °C, 1 min—using the GoTaq Green Master Mix reagent (Promega, Sydney, Australia). Gel electrophoresis was performed with samples run through 1% agarose in 1xTAE buffer and visualised with GelGreen^®^ (Biotium, Fremont, CA, USA).

### 4.3. Screening of Human C2 Expression

Human C2 expression in human or mouse plasma was determined using a human complement C2 ELISA kit according to the manufacturer’s recommendations (RayBiotech Life, Peachtree Corners, GA, USA). Mouse blood from WT, huC2-KI, or a separate colony of C57BL/6J mice was collected into K2 ethylenediaminetetraacetic acid (EDTA) tubes (Sarstedt, Nümbrecht, Germany) and plasma was separated through centrifugation at 2000× *g* for 15 min, 4 °C. Male human plasma was purchased from BioIVT, Woodbury, NY, USA.

### 4.4. Whole-Genome Sequencing

Three male and three female huC2-KI mice were euthanised through CO_2_ inhalation and 10 mg of spleen was collected from each animal and stored at −80 °C. DNA from each splenic sample was prepared using the DNeasy blood and tissue kit (Qiagen, Melbourne, Australia) as per manufacturer’s instructions for tissue samples, and 6 µg from each sample was then sent to Novogene, Singapore for whole-genome sequencing. Sequencing libraries were generated and sequenced on NovaSeq X platform in the 2 × 150 bp configuration. Between 92 Gb and 105 Gb of high-quality sequence, >95% of bases >Q30, were generated per mouse, equivalent to 34× to 39× coverage (based on 2.7 Gb size of GRCm39 mouse reference genome). Reads were aligned with a collection of sequences comprising GRCm39 primary sequences, the human C2 cDNA sequence and the Cre/Neo cassette sequence used in the genetic manipulation. More than 99.8% of the reads were mapped to the reference sequences with <0.5% base mismatch rate and >98% of reads were mapped in proper pairs within expected fragment size distribution.

### 4.5. Complement Inhibition Assays

For the assessment of complement inhibition, the Hycult Biotech Complement Pathway Mouse Assays were used (Hycult Biotech, Uden, The Netherlands). The kits were performed according to the manufacturer’s instructions. In brief, diluted serum samples (murine sera or anti-C2 mAb-spiked serum pools) and control samples were incubated for 1 h at 37 °C on IgM- (classical pathway) or LPS-coated (alternative pathway) microtiter plates. For the lectin pathway assay, the plates were previously activated for 30 min at 37 °C, followed by a washing step. Afterwards, diluted serum samples were incubated for 1 h at 37 °C on Mannan-coated microtiter plates. After washing, a biotinylated tracer antibody (specific for C5b-9) was added to each well and incubated for 1 h at 37 °C. Another washing step was followed by the incubation with a streptavidin–peroxidase conjugate for 1 h at 37 °C. For quantification, a tetramethylbenzidine (TMB) substrate was added. The enzyme reaction was stopped after max. 30 min at room temperature (RT) and absorbance was read at 450 nm with a spectrophotometer. For the calculation of pathway-specific inhibition by CSL305, pre-diluted serum only was set as 100% activity.

### 4.6. Mouse In Vivo Experiments

All animal studies were approved by the Institutional Animal Care and Use Committee, Australia. All animal procedures were conducted in accordance with the ethical standards and guidelines for the care and use of laboratory animals. The study protocols for mouse studies were approved by CSL Animal Ethics Committee #1023, #2024-030 (for anti-GBM studies) and #1065, #2025-011 (for CAIA studies). All efforts were made to minimise animal suffering and to reduce the number of animals used whilst still allowing for appropriate statistical power. C57BL/6J (WT) mice were purchased from Ozgene-ARC, Perth, Australia, and acclimatised to their environment for at least 1 week before the procedures and were allowed food and water ad libitum. huC2-KI mice were bred and housed onsite at the Bio21 Animal Facility, University of Melbourne, Australia. Male WT and huC2-KI mice aged 8- to 9-week-old were used in all studies.

The attenuated passive anti-GBM glomerulonephritis mouse model was performed as previously described [30]. Briefly, the mice were intravenously (IV) injected with 1.0 mg rabbit anti-mouse GBM on day 0. The amount of anti-GBM antibody was below the threshold to induce significant proteinuria in mice. On day 6, the mice were intraperitoneally (IP) injected with 2.0 mg of mouse anti-rabbit IgG mAb (ATCC, Manassas, VA, USA). After mouse anti-rabbit antibody administration, the mice were placed individually in metabolic cages (Tecniplast, Buguggiate, Italy) to collect 24 h urine. Before study commencement, the animals were randomised to treatment groups using an in-house computer-based randomiser, stratified by cage. A group size of *N* = 10 mice was calculated based on the historical data for the primary outcome, the 24 h albuminuria comparisons, to determine drug efficacy. To determine the effect of CSL305 on anti-GBM-induced glomerulonephritis, the mice were IP administered with PBS or CSL305 at the indicated doses, 1 h prior to the injection of mouse anti-rabbit antibody. The measurement of 24 h urine albumin, plasma levels of complement activation and neutrophil kidney infiltration was performed as previously described [30]. No exclusion or blinding was applied in these studies.

Collagen antibody-induced arthritis (CAIA) was induced as previously described [50]. Briefly, on day 0, the animals were IP injected with 2 mg of 5-clone mAb cocktail (Chondrex, Woodinville, WA, USA); on day 3, the mice received IP injection of 50 μg LPS (Chondrex, Woodinville, WA, USA). Evaluation of arthritis development was performed throughout the study (up to 10 days). The severity of arthritis was blindly evaluated in each limb with a scale of 0–3, where 0: no macroscopic signs of arthritis; 0.5: swelling was confined to digits; 1: minor swelling of part of the paws; 2: swelling of entire paw but still flexible; 3: swelling of entire paw with joint stiffness. The maximum score for each animal was 12. To examine the therapeutic effect of CSL305, IP administration at the indicated doses was performed either prophylactically (on day 0 and 3 prior to anti-collagen mAb cocktail and LPS injection, respectively) or therapeutically as a single administration on day 5 when arthritis disease was established. For the prophylactic treatment, the animals were randomised to treatment groups using an in-house computer-based randomiser, stratified by cage. For the therapeutic treatment, the arthritis disease in each animal was blindly scored on day 5 and then the mice were assigned to different treatment groups using stratified randomisation (by cage) based on disease scores. For each animal, two investigators were involved as follows: a first investigator administered the treatment based on the randomisation table. This investigator was the only person aware of the treatment group allocation. The second investigator performed the disease scoring. The mice with body weight loss reaching 25% or greater or 20% over 2 days or those with signs of significant distress after CAIA disease induction were excluded from the studies. The drop-out rate was about 5–10%, which was considered when determining the group size. A priori sample size calculations were performed based on historical data for the primary endpoint, i.e., the total disease scores. The plasma anti-CII antibody levels were measured throughout the studies using an ELISA kit (Chondrex, Woodinville, WA, USA). Assessment of complement activation fragments was performed as described above.

## 5. Conclusions

The in vivo data presented in this study shows that the generation of suitable genetically altered animals such as huC2-KI mice can overcome the difficulties posed by insufficient cross-reactivity of molecules like CSL305 intended for therapeutic use. CSL305 was shown to be both an effective inhibitor of the complement classical and lectin pathways and an effective FcRn antagonist in vivo. Unfortunately, both of the mouse models of disease employed to assess the efficacy of CSL305 fell short of being able to test both mechanisms of action concurrently, rather than separately. Additional mouse models suitable for the huC2-KI strain will need to be identified in order to explore the additive or synergistic effects of CSL305’s dual mechanism in vivo.

## Data Availability

All data are available on request from the authors. Authors confirm that all data are included in the manuscript.

## Abbreviations

FcRn: neonatal Fc receptor; huC2, human C2; KI, knock-in; GBM, glomerular basement membrane; CAIA, collagen autoantibody-induced arthritis; ANCA, anti-neutrophil cytoplasmic antibody; Ig, immunoglobulin; mAb, monoclonal antibody; Fab, fragment antigen binding; Fc, fragment crystallizable; WT, wild-type; cDNA, complementary DNA; PCR, polymerase chain reaction; ELISA, enzyme-linked immunosorbent assay; PBS, phosphate-buffered saline; LPS, lipopolysaccharide; UTR, untranslated region; pA, polyadenylation; EDTA, ethylenediaminetetraacetic acid; TMB, tetramethylbenzidine; RT, room temperature; IV, intravenous; IP, intraperitoneal, anti-CII, anti-collagen type II.
